# First-year medical students’ use of practice questions to understand science courses

**DOI:** 10.3389/fmed.2026.1863776

**Published:** 2026-07-20

**Authors:** Basem Al Nafisah

**Affiliations:** King Abdullah International Medical Research Center, Ministry of National Guard—Health Affairs, King Saud bin Abdulaziz University for Health Sciences, Riyadh, Saudi Arabia

**Keywords:** biomedical science, learning strategies, medical education, practice questions, qualitative research

## Abstract

**Background:**

First-year medical students encounter dense biomedical science content and must develop strategies to achieve conceptual understanding. Practice questions accompanied by explanatory rationales have become widely adopted learning tools, yet how students use these resources to make sense of science course material remains underexplored from a qualitative perspective.

**Aim:**

To explore how first-year medical students use practice questions to understand science course content.

**Methods:**

Semi-structured qualitative interviews were conducted with 15 first-year medical students and analysed using reflexive thematic analysis. For the purposes of this study, practice questions are defined as multiple-choice and case-based items accompanied by detailed explanatory rationales, drawn from both institutional course resources and commercial question banks.

**Results:**

Four themes were constructed: (1) practice questions simplify complex science by breaking content into manageable units; (2) rationales are valued more than answers because they explain underlying concepts; (3) questions are used to confirm understanding rather than initially build it; and (4) questions are often treated as single-use learning tools once understanding feels secure.

**Conclusion:**

Practice questions function as meaning-making tools for first-year medical students navigating science courses. Students prioritise explanatory rationales over correct answers and integrate questions into routines that serve both constructive and confirmatory purposes. These findings highlight the importance of recognising how students engage with learning resources in science-heavy curricula.

## Introduction

Medical education assumes that students develop a robust understanding of biomedical science as a foundation for clinical reasoning and professional practice (1). The ability to understand complex scientific concepts—rather than merely memorise them—is essential for physicians who must apply knowledge flexibly across varied clinical contexts throughout their careers (2, 41). Yet first-year medical students frequently struggle to grasp the relevance and meaning of dense biomedical content, particularly when confronted with abstract principles and unfamiliar terminology (3). This challenge is compounded by the volume and pace of preclinical science courses, which demand that students rapidly develop effective learning approaches (4). Early difficulty in achieving conceptual understanding can shape students’ confidence and engagement with foundational sciences in ways that persist well beyond the first year (5). Understanding how students navigate this challenge is therefore both an educational and a professional concern.

To manage the demands of biomedical science courses, students actively select learning tools they perceive as helpful for achieving clarity and understanding. Study strategy preferences in early medical training are diverse and reflect individual judgements about what supports learning most effectively (6). Research suggests that self-directed strategy choices vary in effectiveness depending on the learner’s metacognitive awareness and the alignment between strategy and learning goal (7); not all choices produce equivalent outcomes. Students often choose resources based on their perceived level of preparedness and the extent to which a tool reduces uncertainty (8). Stress and workload further influence these decisions, as students seek efficient approaches that provide reassurance while managing competing time demands (9, 10). From an adult learning perspective, medical students are self-directing learners who select strategies based on prior experience and perceived usefulness (11–13, 39). Recognising students as active decision-makers in their own learning is therefore central to understanding how they engage with educational resources.

Practice questions have become a prominent feature of the digital learning environment in medical education. As defined above, practice questions here refer to multiple-choice and case-based items accompanied by explanatory rationales that clarify why answer options are correct or incorrect—resources that extend beyond simple recall testing to support conceptual understanding (14–16). Many preclinical programmes now provide students with early access to question banks designed to support formative learning alongside course content (17, 37). Early engagement with board-style questions from commercial platforms has been associated with improved examination performance and perceived preparedness (18). Such platforms have been integrated into a range of medical training contexts, functioning as both assessment preparation tools and learning supports (19, 20). Together, these digital resources form part of the e-learning ecosystem that increasingly mediates how students interact with course material (21). Qualitative research has, however, also identified potential pitfalls of question bank use—including cueing, avoidance, and surface-level memorising—that may undermine the development of genuine understanding (22).

Research on retrieval practice has established that testing can support long-term retention when used strategically (23), and more recent work confirms that repeated retrieval with sufficient spacing prevents knowledge loss across preclinical science domains (24). Yet qualitative insight into how first-year medical students engage with practice questions in day-to-day learning—particularly when their goal is conceptual understanding rather than assessment preparation—remains limited. Students modify their learning strategies across the first year as they adapt to new academic demands (25), and self-directed learning behaviours vary widely across individuals (26). Qualitative work on how students engage with feedback-bearing assessments—including progress tests and formative question tools—further suggests that access to good feedback does not automatically translate into productive use of it (27). This study addressed the gap by exploring how first-year medical students use practice questions to understand science course content.

## Methods

This qualitative study used semi-structured interviews to examine students’ experiences and perspectives. Participants were first-year medical students enrolled in preclinical science courses including anatomy, physiology, and biochemistry, recruited through email invitations and course announcements. Interviews took place during the second semester of the first year, after students had sufficient exposure to preclinical science content and practice question resources to speak meaningfully about their experiences, but before end-of-year summative examinations to reduce recall bias. Purposive sampling was used to capture variation in prior academic background including public high school versus private high school graduate and diverse academic performance. Recruitment continued until thematic saturation was reached, consistent with contemporary guidance on assessing saturation in qualitative research (28, 29). Saturation was determined iteratively: after each interview, new transcripts were reviewed against the emerging code set, and recruitment ceased when no new codes or conceptual categories appeared across three consecutive interviews. Fifteen participants were interviewed in total.

Data were collected through individual semi-structured interviews of approximately 45–60 min. Students drew on practice questions from two sources: institutional question sets provided within the preclinical curriculum, and commercial question banks (primarily AMBOSS and UWorld) that participants reported accessing independently; no single resource type was prescribed. Although the specific content of individual questions and rationales varied across platforms, all shared the same core structure: a stem question followed by answer options and an explanatory rationale. Institutional resources tended toward shorter, factual stems aligned with weekly lecture content; commercial questions used longer case-vignette formats. Both types were reported as useful, though several participants noted that the narrative depth of commercial questions was particularly helpful for understanding mechanistic connections.

The interview schedule addressed how students used practice questions, what features they valued in these resources, and how practice questions supported their understanding of science content. Interviews were conducted by the author, who had no direct teaching relationship with participating students at the time of data collection, minimising the potential for social desirability effects. All interviews were audio-recorded and transcribed verbatim. Core prompts included: (1) ‘Can you walk me through how you typically use practice questions when studying?’; (2) ‘What do you find most useful about the explanatory rationales?’; (3) ‘Do you revisit questions you have already completed, and why or why not?’.

Data analysis followed the reflexive thematic analysis approach described by Braun and Clarke (30, 31). The analytic process involved repeated familiarisation with the transcripts, systematic coding, development of initial themes, and iterative refinement. An inductive approach was used: codes and themes were generated from participants’ accounts rather than from a predefined framework. To support transparency and trustworthiness, the author maintained a reflexive journal throughout analysis, and a sample of transcripts was independently coded by a second researcher; discrepancies were resolved through discussion until consensus was reached. As this study was wholly qualitative in design, no statistical methods were employed.

The study received institutional ethics approval prior to data collection. All participants provided written informed consent, were assured of confidentiality, and were informed that participation was voluntary and would not affect their academic standing.

## Results

Four themes were constructed from the analysis. The large majority of participants described the patterns captured in Themes 1, 2, and 3, reflecting their centrality to how students approached practice questions.

Theme 4 was reported by approximately half the sample. These students described a consistent pattern: once they had worked through a question, read the rationale, and felt they understood the underlying concept, they moved on permanently. The same question would not be revisited. The same topic would not be returned to unless a new question on it appeared in the natural course of forward progress through the material. Understanding, once felt, was treated as complete.

What made this subgroup particularly notable was that several of them demonstrated awareness that their approach was at odds with evidence on how learning actually works. These students had encountered—through their curriculum, their own reading, or peer conversations—the idea that repeating questions and spacing out retrieval produces better long-term retention than a single exposure. They could articulate this. They were not operating from ignorance of better strategies. And yet they continued with single-use engagement anyway, citing the sheer volume of preclinical content and the pressure of keeping pace with the curriculum as reasons why revisiting felt like a luxury they could not afford.

This tension is educationally significant for two reasons. First, it reveals that the barrier to spaced retrieval in this population is not primarily a knowledge gap—students know the recommendation—but a perceived cost–benefit problem. Repeating completed questions feels like lost time when there is always new material ahead. Second, it suggests that awareness of learning science principles alone is insufficient to change behaviour. Students can hold the correct belief about what works while still choosing a strategy that conflicts with it, because the immediate demands of the curriculum make the efficient choice feel more rational than the effective one. The gap these students were describing is not between ignorance and knowledge—it is between knowledge and action, and that is a harder gap to close.

A sequential pattern was also apparent across participants who drew on more than one theme. Students typically engaged with Theme 1 (simplifying) early in a study session, moved to Theme 3 (confirming) once initial reading was complete, and drew on Theme 2 (rationale engagement) throughout both stages. Theme 4 (single-use) operated as a termination decision—students chose not to revisit material once Theme 3 was satisfied—rather than as a discrete step in the learning process. This sequence was not universal but describes the modal pattern in the data and is presented here to clarify how the themes relate to one another in practice.

### Theme 1: practice questions simplify complex science

Students described practice questions as breaking dense biomedical content into manageable, focused units. When faced with lengthy textbook chapters or lecture slides covering multiple interconnected concepts, students found questions useful for isolating specific ideas and working through them systematically. Questions imposed structure and boundaries on material that otherwise felt boundless, and students valued how they translated broad scientific principles into concrete scenarios that demanded active engagement with a specific idea. This process of working through individual questions helped students organise and clarify their understanding of content that would otherwise have felt overwhelming.

Some students reported initial difficulty using questions productively in the early weeks of the course. They described a period of adjustment: questions felt frustrating before they had accumulated sufficient background knowledge to engage meaningfully with the rationales. This threshold effect reflects a dependency that shapes the practical usefulness of question-based resources—they function as simplifying tools only once students have enough contextual knowledge to make sense of the explanations offered. As students built that base over the early weeks, questions became progressively more productive as structuring devices.

*"When I look at a whole lecture on renal physiology it feels like too much. But when I do a question on tubular reabsorption, it forces me to focus on just that piece and understand it before moving on. (P3)"*

*"The questions break it down for me. Instead of reading ten pages and feeling lost, I do a question and I know exactly what I need to understand. (P9)"*

### Theme 2: rationales valued more than answers

Students consistently emphasised that the explanatory rationales accompanying practice questions were more valuable than the answers themselves. Rationales clarified why answers were correct, explained the underlying mechanisms, and addressed common misconceptions. Many students described reading rationales even for questions they answered correctly, using the explanation to deepen their understanding or to encounter alternative ways of thinking about the material. Rationales functioned as compact teaching moments that reinforced or extended conceptual grasp—valued particularly when they connected ideas across topics, traced reasoning pathways, and explained not just which option was correct but why the other options were wrong.

This emphasis on explanation over answer shaped how students engaged with questions throughout their study sessions. The resources available to these students—institutional rationales and commercial question bank explanations—were specifically designed to foreground conceptual explanation. The design of these resources may therefore have reinforced the meaning-oriented engagement observed here; students using resources with minimal or absent rationales may engage quite differently. This possibility is consistent with qualitative evidence that the structural features of assessment tools—particularly the presence and quality of explanatory feedback—significantly shape how students process and act on results (27).

*"I don't even care about the answer most of the time. I read the rationale even when I get it right because that's where the actual explanation is. (P1)"*

*"The rationale tells me why the other choices are wrong, and that's what actually teaches me the concept. Just knowing the right answer doesn't help me understand. (P12)"*

### Theme 3: questions used to confirm understanding

Students used practice questions as comprehension checkpoints rather than as primary learning activities. After reviewing lecture materials or assigned readings, they turned to questions to verify whether they had genuinely understood the content or had merely followed the words on the page. Questions served as external evidence that learning had occurred: correct performance with explained reasoning provided reassurance, while difficulty signalled a need to revisit the material or seek further explanation.

This confirmatory use meant that practice questions were embedded in study routines after—not instead of—initial content engagement. Students framed this as an active strategy for managing uncertainty, using question performance as a more objective check on their sense of readiness rather than relying on subjective feeling alone. This reflects a degree of metacognitive monitoring, though its reliability warrants scrutiny. Research on metacognition in pre-clinical medical students indicates that students at this stage often rely on superficial predictive cues when assessing their own knowledge, and that explicit metacognitive skills are underdeveloped and require deliberate teaching to improve (32). Confident performance on a practice question may therefore reflect familiarity with the item format or the particular question encountered, rather than robust command of the underlying concept.

*"I read through the lecture notes first, then I do questions to check if I actually understood it or if I was just reading words. If I get a question wrong, I know I need to go back. (P7)"*

*"For me the question is almost like a test of my own understanding. It's not how I learn—it's how I confirm what I already learned. (P4)"*

### Theme 4: questions treated as single-use learning tools

Many students described treating practice questions as single-use resources: once a question’s rationale made sense and they felt they had grasped the underlying principle, they moved on and did not return to the same item. Efficiency and forward coverage were the stated priorities. Revisiting a question that had already yielded understanding seemed redundant, and several students acknowledged that they were aware of evidence recommending repeated retrieval but found it difficult to act on given the volume of content and time pressures of the preclinical curriculum.

This pattern raises a concern that the sense of understanding students describe may be more transient than durable. Following a rationale at the moment of study does not guarantee that the concept can be retrieved later under examination or clinical conditions—a distinction that students appeared largely unaware of when describing their strategies. This gap is consistent with what has been termed the ‘illusion of knowing’: a transient sense of competence at the point of study that does not persist as retrieval-ready knowledge (33). Empirical studies of preclinical medical students confirm that content covered without subsequent retrieval practice shows significant knowledge decay within weeks, even when students report feeling confident at the time of study (24). Single-use engagement, however efficient it feels in the moment, may therefore contribute to the well-documented gap between subjective confidence and objective retention (23, 24).

Several participants also described refining their approach across the semester: initially attempting questions before adequate preparation and finding them unhelpful, then progressively shifting to a post-study confirmatory pattern as they developed a clearer sense of what prior knowledge a question required. This adaptive trajectory is evidence of some metacognitive monitoring, even if students’ conclusions did not always align with evidence-based recommendations. Similar iterative strategy revision has been documented among medical students in both preclinical and clinical learning contexts (25).

*"Once I understand why the answer is what it is, there's no point doing the same question again. I'd rather do a new question on a topic I haven't covered yet. (P2)"*

*"I know they say you should repeat questions, but honestly there's just not enough time. I need to keep moving forward. (P11)"*

## Discussion

This study supports the view that first-year medical students use practice questions as tools for meaning-making rather than purely as assessment preparation or retrieval devices. Students described engaging with questions to actively construct understanding of abstract biomedical concepts—not merely to test recall. This extends prior work documenting students’ difficulties in perceiving the relevance of biomedical science during early medical training (3): whereas those studies identified the problem, the present findings illustrate one way in which students attempt to resolve it. By breaking complex material into focused units and providing explanatory frameworks, practice questions help students achieve a working grasp of dense scientific content. This meaning-making orientation is consistent with Sheehy et al. (15), who similarly observed that medical students valued practice questions primarily as active learning supports rather than as vehicles for assessment rehearsal. It should be acknowledged, however, that this study did not assess learning outcomes directly; perceived conceptual gains may not always translate to durable or retrievable knowledge.

Students’ emphasis on rationales over correct answers highlights the central role of explanation in their learning process. Rather than treating questions as performance tests, students described rationales as compact teaching moments that clarified misconceptions and deepened their conceptual models. This is consistent with research showing that quiz-based activities are most educationally effective when they include explanatory feedback (14), and with the broader recognition that formative tools embedding detailed explanation serve learning functions that extend beyond their summative or evaluative role (38). The present findings suggest that students use rationales to monitor and regulate their understanding (34)—seeking confirmation that their internal model of a concept is accurate before proceeding. This monitoring function is especially important in science-heavy courses where unfamiliar vocabulary, abstract mechanisms, and layered concepts must be integrated simultaneously. The work of van Wijk et al. (27) on progress test feedback is instructive here: even when feedback is well-structured and readily available, students often cannot translate it into actionable learning strategies without metacognitive scaffolding. By extension, access to a well-written rationale does not automatically ensure that students extract, consolidate, or later retrieve the understanding it offers—an important caution for those who assume that rich explanations are sufficient on their own.

The finding that students often treat practice questions as single-use resources offers insight into how they balance efficiency with learning depth. Once a concept feels understood, repeating the same question seems unnecessary—particularly given the volume of content and time pressures of the preclinical curriculum. This approach contrasts with a substantial body of evidence showing that spaced, repeated retrieval is among the most effective strategies for long-term knowledge retention (23, 24). Single-use engagement may partly reflect an ‘illusion of knowing’ (33): the fluency that comes from reading a clear rationale can be mistaken for retrievable knowledge, making revisiting feel redundant to students who already feel they understand. For students at the early stages of clinical training—whose foundational science knowledge must remain accessible and applicable across years of study and practice—this gap between perceived and actual learning carries meaningful consequences. Evidence from a study of preclinical medical students confirms that topics not revisited through spaced retrieval show substantially greater knowledge decay than those encountered across multiple sessions (24). Students’ single-use pattern may also reflect cognitive load management: once a concept feels resolved, allocating attention to new material is the rational short-term choice (35), even if it is not optimal for long-term retention.

These findings have implications for how question-based resources are designed and integrated into curricula. Students are not passive consumers of question banks: they bring specific goals—clarity, reassurance, reduced uncertainty—and they select and use resources accordingly (8). Fisher et al. (22, 40) found that early-stage medical students using question banks were predominantly driven by extrinsic motivation, exhibiting patterns of cueing and surface memorising that may undermine genuine understanding. The contrast with the more meaning-oriented engagement described in the present study suggests that the goal orientation students bring to question use may be as consequential as the design of the resource itself. Where students are primarily motivated by fear of failure rather than intrinsic interest in understanding, even rich explanatory rationales may be read for the correct answer rather than for the reasoning behind it. This points to the value of educators explicitly discussing with students what productive engagement with question rationales looks like and why single-use engagement is likely insufficient for durable retention. Educators should also consider that the engagement patterns observed here may be specific to contexts where resources foreground explanation; programmes relying on question banks with minimal or formulaic rationales may see quite different student behaviour.

A final consideration concerns the institutional and cultural context of this study. The research was conducted in a single context, where the structure of medical education, cultural orientations toward self-directed and collaborative learning, and prevailing norms around academic resource use may differ meaningfully from settings in other international contexts. Research on learning approaches in preclinical medical students has found that curricular model, the presence of collaborative learning structures, and the degree of learner autonomy all shape whether students adopt deep or surface approaches (36). These contextual factors should be considered carefully when applying the present findings to other settings. The patterns described here are best understood as a situated account of how one group of students made sense of a specific learning environment—and the degree to which they generalise, and under what conditions, is itself a productive question for future comparative research.

### Limitations

Several limitations warrant acknowledgement. First, the study recruited from a single institution; the cultural context, curricular structure, and resource landscape may limit the transferability of findings to other settings. Second, the sample of 15 participants, while adequate for thematic saturation in this focused inquiry, represents a narrow slice of the first-year medical student population. Third, all data are self-reported: students described their practices retrospectively in interviews, which introduces the risk of recall bias and social desirability effects despite the interviewer’s precautions. Fourth, learning outcomes were not assessed; the themes reflect students’ perceptions and descriptions of their engagement, not evidence that the engagement produced measurable conceptual understanding or retention. Fifth, data were collected during a single semester, providing a cross-sectional snapshot; longitudinal tracking would be necessary to understand how strategies evolve across the full preclinical period and into clinical training. Taken together, these constraints bound what can be claimed from the findings, and readers should weigh them accordingly when considering how the results apply to their own contexts.

## Conclusion

First-year medical students use practice questions as meaning-making tools to understand complex biomedical science content. They value explanatory rationales that clarify concepts and address misconceptions, use questions to confirm rather than initially build understanding, and often treat questions as single-use resources once comprehension feels secure. A sequential pattern was also apparent, with simplifying and confirming functions operating at distinct points in the study session and rationale engagement running throughout. These findings underscore the importance of recognising how students actively engage with learning tools in science-heavy curricula. Students would benefit from explicit guidance on the distinction between the transient clarity a rationale produces at the point of study and the durable retrieval that comes only from repeated, spaced engagement with material. Curriculum designers may consider making high-quality explanatory rationales a central and visible feature of question-based resources, and educators may find it productive to incorporate structured discussion of evidence-based study strategies—particularly spaced retrieval—into early preclinical teaching. Future research should examine how engagement patterns vary with question format, rationale quality, and institutional context, and should track how practice question use evolves as students progress through preclinical and into clinical training.

## Data Availability

The original contributions presented in the study are included in the article, further inquiries can be directed to the corresponding author.
